# Coating of Intestinal Anastomoses for Prevention of Postoperative Leakage: A Systematic Review and Meta-Analysis

**DOI:** 10.3389/fsurg.2022.882173

**Published:** 2022-04-22

**Authors:** Kamacay Cira, Felix Stocker, Stefan Reischl, Andreas Obermeier, Helmut Friess, Rainer Burgkart, Philipp-Alexander Neumann

**Affiliations:** ^1^ Department of Surgery, School of Medicine, Klinikum rechts der Isar, Technical University of Munich, Munich, Bavaria, Germany; ^2^ Institute of Diagnostic and Interventional Radiology, School of Medicine, Klinikum rechts der Isar, Technical University of Munich, Munich, Bavaria, Germany; ^3^ Department of Orthopaedics and Sports Orthopaedics, School of Medicine, Klinikum rechts der Isar, Technical University of Munich, Munich, Bavaria, Germany

**Keywords:** intestinal anastomoses, coated collagen patch, fibrin sealant, fibrin glue (FG), anastomotic leakage (AL)

## Abstract

**Background:**

For several decades, scientific efforts have been taken to develop strategies and medical aids for the reduction of anastomotic complications after intestinal surgery. Still, anastomotic leakage (AL) represents a frequently occurring postoperative complication with serious consequences on health, quality of life, and economic aspects. Approaches using collagen and/or fibrin-based sealants to cover intestinal anastomoses have shown promising effects toward leak reduction; however, they have not reached routine use yet. To assess the effects of covering intestinal anastomoses with collagen and/or fibrin-based sealants on postoperative leakage, a systematic review and meta-analysis were conducted.

**Method:**

PubMed, Web of Science, Cochrane Library, and Scopus (01/01/1964 to 17/01/2022) were searched to identify studies investigating the effects of coating any intestinal anastomoses with collagen and/or fibrin-based sealants on postoperative AL, reoperation rates, Clavien–Dindo major complication, mortality, and hospitalization length. Pooled odds ratios (ORs) with 95% confidence intervals (CIs) were calculated.

**Results:**

Overall, 15 studies (five randomized controlled trials, three nonrandomized intervention studies, six observational cohort studies) examining 1,387 patients in the intervention group and 2,243 in the control group were included. Using fixed-effects meta-analysis (*I*^2^ < 50%), patients with coated intestinal anastomoses presented significantly lower AL rates (OR = 0.37; 95% CI 0.27–0.52; *p *< 0.00001), reoperation rates (OR, 0.21; 95% CI, 0.10–0.47; *p *= 0.0001), and Clavien–Dindo major complication rates (OR, 0.54; 95% CI, 0.35–0.84; *p *= 0.006) in comparison to controls, with results remaining stable in sensitivity and subgroup analyses (stratified by study design, age group, intervention used, location of anastomoses, and indication for surgery). The length of hospitalization was significantly shorter in the intervention group (weighted mean difference (WMD), −1.96; 95% CI, −3.21, −0.71; *p *= 0.002) using random-effects meta-analysis (*I*^2 ^≥ 50%), especially for patients with surgery of upper gastrointestinal malignancy (WMD, −4.94; 95% CI, −7.98, −1.90; *p *= 0.001).

**Conclusion:**

The application of collagen-based laminar biomaterials or fibrin sealants on intestinal anastomoses can significantly reduce postoperative rates of AL and its sequelae. Coating of intestinal anastomoses could be a step toward effective and sustainable leak prevention. To assess the validity and robustness of these findings, further clinical studies need to be conducted.

## Introduction

In the field of visceral surgery, both patients and surgeons are still challenged with a very common and potentially devastating postoperative complication, namely, anastomotic leakage (AL). Whether the intestinal anastomoses were performed in the upper or lower gastrointestinal tract (GIT), postoperative AL accompanies a significant proportion of intestinal surgical procedures (1–10). Colorectal procedures, for instance, present with AL rates of up to 25.6% (4, 8, 9), and esophageal or esophagogastric procedures present with AL rates as high as 19.5% (6, 7). AL rates among patients with malignancies are even associated with local (11) and distant (12) tumor recurrences. Furthermore, AL has been shown to increase the total clinical and economic burden by 0.6–1.9 times for patients undergoing intestinal surgery for colorectal cancer (13).

In this context, it is not surprising that substantial scientific efforts have been invested now for over half a century to develop strategies and medical aids to reduce or even prevent the development of postoperative AL. The first approach toward covering and hereby mechanically strengthening the newly built intestinal anastomosis was to apply cyanoacrylate preparations, better known as surgical glues initially tested on skin wounds in military settings. Their rapid formation of a stable but flexible connection with intestinal tissue was considered advantageous (14, 15). Other experimental approaches utilized sterile polyethylene plastic sheets (16), fibrin adhesives (17, 18), and collagen fleeces (17) to additionally support the anastomoses. The most promising adhesives, however, are fibrin sealants, as these have been acknowledged across various surgical specialties and were approved in their liquid form by the FDA in 1998 (19).

Biodegenerable and absorbable fibrin sealants consist of two components: sealer protein solution (human fibrinogen, factor XIII, and protease inhibitor aprotinin) and thrombin solution (human thrombin and calcium chloride). Upon application of the sealant to the site of anastomosis, thrombin transforms fibrinogen into insoluble fibrin monomers, which are then polymerized in the presence of factor XIII to a stable fibrin network within minutes. Protease inhibitor aprotinin protects this network from plasmin-mediated proteolysis. Simulating the last step of the coagulation cascade, fibrin sealants are used to initiate hemostasis, seal tissue, and promote the healing processes (20).

With the 2010 FDA approval of a fibrin sealant-coated equine collagen matrix (21) used primarily for hemostatic purposes, experimental approaches studying its potentially beneficial effect on anastomotic healing were initiated. Within the last decade, mainly animal studies were conducted, revealing promising effects on reducing postoperative AL and mortality rates upon using either fibrin sealants or collagen-based laminar biomaterials (22–33). For many years, just a small number of experimental trials have been available, examining the effect of these sealants on human populations (34–40). Until now, no meta-analysis has been conducted examining the effect of externally covering intestinal anastomoses with collagen-based laminar biomaterials or fibrin sealants on postoperative AL and its consequences within a human population.

Therefore, the aim of this study was to systematically evaluate the efficacy of externally coating intestinal anastomoses of the upper and lower GIT, regardless of location or underlying disease, with collagen-based laminar biomaterials and/or fibrin sealants in reducing postoperative AL rates and its accompanying complications. A systematic review and meta-analysis of existing human studies was conducted, comparing the summary effect size, calculating the pooled odds ratios (ORs) with 95% confidence intervals (CIs), and performing subgroup analyses stratified by study design, coating utilized, age group, indication for surgery, and location of anastomoses.

## Methods

This systematic review and meta-analysis was conducted and reported according to the recommendations in the *Cochrane Handbook for Reviews of Interventions* (41) and the *Preferred Reporting Items for Systematic Review and Meta-Analyses* (*PRISMA*) *Statement 2020* (42).

### Eligibility Criteria

For this study, all observational studies (prospective or retrospective comparative cohort or case–control studies), nested case–control studies, randomized controlled trials (RCTs), nonrandomized controlled trials, and cross-sectional studies were included based on the following criteria: examined a human population—regardless of age, sex, or underlying condition; published only in English, German, or Spanish language; available as either abstract or full-text article in the medical databases between 01/01/1964 and 17/01/2022; included humans undergoing any intestinal surgical procedure with the formation of any kind of intestinal anastomoses with focus on the upper and lower GIT; the intervention group included patients who received an intestinal anastomosis (regardless of anastomotic technique) coated or reinforced with either a collagen-based laminar biomaterial or a fibrin sealant (synthetic or animal derived, with or without additional substances embedded, regardless of the manufacturer); control group included patients who received an intestinal anastomosis (regardless of anastomotic technique) not coated or reinforced with any product; and depicted postoperative clinical outcomes, including but not restricted to, AL, reoperation and mortality rates, major complication rates (grades III–V) according to the Clavien–Dindo classification of surgical complications (43) (C-DMC), and length of hospital stay.

Exclusion criteria comprised studies representing reviews or meta-analyses, case reports or case series, animal studies, *ex vivo* or *in vitro* studies; gastrointestinal surgical procedures without the formation of an intestinal anastomosis; hepatobiliary anastomoses (e.g., pancreaticointestinal anastomoses, biliodigestive anastomoses); closure of transmural and nontransmural intestinal defects; intestinal stumps or pouches; coating of anastomosis in an operative revision, secondary to AL or fistula formation; and any kind of anastomotic coatings or sealants not based on collagen and/or fibrin.

### Search Strategy

We conducted a comprehensive systematic literature search for studies published in the electronic medical databases PubMed (MEDLINE), Web of Science, Scopus, and Cochrane Library using predefined search items, further specified in Supplementary Table S1.

To ensure that potentially relevant studies were not missed, reference lists of reviews and included studies were examined manually, and additional web search was conducted. In case of ambiguous or inadequate data presentation, we contacted these studies’ authors to provide the required information. The final search was conducted on 17/01/2022.

### Selection Process

Study selection was performed by two investigators (surgical residents: K.C. and F.S.) independently. All studies identified in the search process were exported to the reference management tool EndNote X9 (The EndNote Team, Clarivate 2013, Philadelphia, PA, USA).

Duplicates were removed by computer-based methods, followed by a secondary manual exclusion. Titles and abstracts were assessed manually and excluded in accordance with our predefined eligibility criteria. Abstracts and full-text articles correlating with these criteria were retrieved and further evaluated for eligibility. Disagreements concerning eligibility were discussed and resolved in consensus with a third investigator (surgical specialist: P-A.N.), who independently assessed the accuracy of the search results.

### Data Collection Process

Two investigators (K.C. and F.S.) independently performed data collection and analysis onto a Microsoft Excel spreadsheet (Home and Student 2019 edition; Microsoft, Redmond, WA, USA), and a third investigator (radiology resident: S.R.) independently assessed the accuracy of the extracted data. In case of any discrepancies, the extracted data were discussed and resolved in consensus with the fourth investigator (P-A.N.) acting as an arbitrator.

### Data Extraction

For each study, we collected the following data, if available: author, year, and country of publication; study design and inclusion period; ethical approval and funding; inclusion and exclusion criteria; number of patients in the intervention and control group; baseline characteristics such as age, sex, and body mass index; surgical characteristics: indication for surgery, surgical intervention and technique, and anastomoses (number, location, and technique); collagen-based biomaterial or fibrin sealant used in the intervention group; and any additional intervention. Study and patients’ characteristics are presented in **Table 1**, and surgical characteristics are given in **Table 2**.

**Table 1:** Study and patient characteristics.

Author	Year	Country	Study design	Age group	Number of patients, *n*		Anastomotic coating (intervention group)	Indication for surgery
					I	C	
Brehant et al. (50)	2013	France	RCS	Adult	202	404	Collatamp Sponge (C-BLB)	Colorectal cancer; Benign lesions
Marano et al. (54)	2016	Italy	RCS	Adult	28	34	TachoSil (C-BLB)	Gastric cancer; Esophagogastric junction cancer
Torres-Melero et al. (58)	2016	Spain	NRS	Adult	22	27	Fibrin-coated collagen sponge (C-BLB)	Peritoneal carcinomatosis (colorectal cancer)
Fernandez et al. (34)	1996	Spain	RCT	Adult	42	44	Tissucol (FS)	Gastric adenocarcinoma
Grieder et al. (51)^a^	2010	Switzerland	Pilot-study	Adult	118	113	Fibrin Glue (FS)	Colorectal cancer
Huang et al. (52)	2021	China	RCS	Adult	86	141	Bioseal (FS)	Squamous cell or adenocarcinoma of the thoracic or esophagogastric junction
Huh et al. (36)	2010	Korea	PCS	Adult	104	119	Tissucol or Greenplast (FS)	Rectal cancer
Kim et al. (53)	2013	Korea	RCS	Adult	414	734	Tissucol or Greenplast (FS)	Rectal cancer
Liu et al. (37)	2003	United States	NRS	Adult	120	360	Tisseel (FS)	Obesity (bariatric surgery)
Oliver et al. (55)	2012	Spain	RCT	Adult	52	52	Tissucol Duo (FS)	Different conditions (high-risk anastomoses)
Saldaña-Cortés et al. (38)	2009	Mexico	NRS	Pediatric	14	24	Quixil (FS)	Caustic esophageal injury
Sdralis et al. (56)	2019	Greece	RCT	Adult	35	22	Tisseel (FS)	Adenocarcinoma of the distal esophagus or esophagogastric junction
Sieda et al. (57)	2015	Egypt	PCS	Adult	35	35	Commercial Fibrin Sealant	Malignant colonic obstruction; Nonmalignant colonic obstruction
Silecchia et al. (39)	2006	Italy	RCT	Adult	93	111	Tissucol (FS)	Obesity (bariatric surgery)
Upadhyaya et al. (40)	2007	India	RCT	Pediatric	22	23	Tisseel (FS)	Esophageal atresia with tracheoesophageal fistula

*RCS, Retrospective cohort study; PCS, prospective cohort study; RCT, randomized controlled trial; NRS, nonrandomized study*; *I, Intervention group (coated or reinforced anastomoses); C, control group*; *C-BLB, collagen-based laminar biomaterial; FS, fibrin sealant; Benign lesions*, *diverticulitis, inflammatory bowel disease, or other lesions; nonmalignant colonic obstruction*, *perforated diverticulum, inflammatory bowel disease, volvulus, fecal fistula, bands.*

*^a^* *Abstract.*

**Table 2:** Surgical characteristics.

Author	Year	Open/laparoscopic^b^	Surgical intervention	Site and technique of anastomosis	
				I^c^/C^c^	Anastomotic covering/reinforcement (I^c^)
Brehant et al. (50)	2013	✓/✓	Colon or colorectal resection	Intestinal anastomosis	Collatamp (10 × 10 cm)
Marano et al. (54)	2016	✓/—	Total or distal gastric resection; Distal esophagectomy and total gastrectomy	Mechanical end-to-side esophagojejunal anastomosis (25 mm anvil head circular stapler); mechanical side-to-end gastrojejunal anastomosis (28 mm anvil head circular stapler)	TachoSil (9.5 × 4.8 × 0.5 cm with two seromuscular stitches)
Torres-Melero et al. (58)	2016	N/A	Debulking colon resection	Mechanical intestinal anastomosis	Fibrin-coated collagen sponge (9.5 × 4.8 cm)
Fernandez et al. (34)	1996	N/A	Curative R_2_ or extended gastrectomy	Mechanical end-to-side esophagojejunal anastomosis (Roux-en-Y jejunal loop used; tobacco pouch formed manually)	Tissucol (applied on both surfaces during approximation of anvil to the Stapler Cartridge)
Grieder et al. (51)^a^	2010	✓/✓	Colorectal resection	Mechanical intestinal anastomosis (approximately 10 cm above anal verge)	Fibrin glue (1 mL; applied between pressure plates of stapler, fired after 2–3 min)
Huang et al. (52)	2021	✓/✓	McKeown esophagectomy	Mechanical end-to-side esophagogastric anastomosis (inverted; circular stapler: EEA 21 or 25 mm)	Bioseal (2.5 mL)
Huh et al. (36)	2010	—/✓	Low anterior rectal resection	Double-stapled colorectal anastomosis	Tissucol or Greenplast (1–2 mL)
Kim et al. (53)	2013	✓/✓	Low anterior rectal resection with total mesorectal excision	Double-stapled colorectal anastomosis	Tissucol or Greenplast (1–2 mL)
Liu et al. (37)	2003	✓/✓	Roux-en-Y-gastric bypass	Hand-sewn gastrojejunal anastomosis	Tisseel (5 mL; perivisceral fat pad glued to anterolateral part of anastomosis)
Oliver et al. (55)	2012	N/A	Esophageal resection; Roux-en-Y-gastric bypass; gastrectomy; rectal resection; intestinal resection of obstructed segment	Intestinal anastomosis (according to procedure)	Tissucol
Saldaña-Cortés et al. (38)	2009	✓/−	Colon interposition for esophageal reconstruction	Hand-sewn, single layer, end-to-side cervicocolic anastomosis covered (4-0 Vicryl)	Quixil (3–4 mL)
Sdralis et al. (56)	2019	✓/✓	Two-stage esophagectomy—Ivor-Lewis procedure	Intrathoracic mechanical end-to-side esophagogastric anastomosis (circular stapler: CDH 25 OR 29 mm)	Tisseel
Sieda et al. (57)	2015	✓/—	Enterocolic resection or colectomy	Hand-sewn, single layer, enterocolic or colocolic anastomosis (continuous suture, 3-0 Vicryl)	Fibrin sealant
Silecchia et al. (39)	2006	✓/—	Roux-en-Y-gastric bypass	Mechanical or hand-sewn gastrojejunal anastomosis (Gagner technique with circular stapler 25 EEA; linear stapler; two-layer continuous suture); jejunal anastomosis	Tissucol (2- or 5-mL)
Upadhyaya et al. (40)	2007	✓/—	Esophageal reconstruction	Hand-sewn, single layer, end-to-side esophageal anastomosis (5-0 Vicryl)	Tisseel

*N/A, Not available; mm, millimeter; cm, centimeter; mL, milliliter;* ✓, *yes; –*, *no; I*, *intervention group (coated or reinforced anastomoses); C = Control Group.*

*^a^* *Abstract.*

To provide an implication on and utilization in surgical practice, data on postoperative AL, reoperation, C-DMC, mortality rate, and the length of hospitalization were collected (**Table 3**).

**Table 3:** Postoperative outcomes.

Author	Year	Anastomotic leakage, *n* (%)		Reoperation, *n* (%)		Clavien-Dindo major complications (43), *n* (%)		Length of hospitalization, mean (SD)^b^; in Days		Mortality, *n* (%)	
		**I** ^c^	**C** ^c^	**I** ^c^	**C** ^c^	**I** ^c^	**C** ^c^	**I** ^c^	**C** ^c^	**I** ^c^	**C** ^c^
Brehant et al. (50)	2013	N/A	N/A	N/A	N/A	**↓18 (9)**	**↑67 (16.6)**	**↓**	**↑**	N/A	N/A
Marano et al. (54)	2016	0 (0)	4 (11.8)	N/A	N/A	N/A	N/A	**↓ 14.7 ± 4.3**	**↑ 19.9 ± 5.6**	0 (0)	0 (0)
Torres-Melero et al. (58)	2016	0 (0)	3 (11.1)	1 (4.6)	3 (11.1)	N/A	N/A	N/A	N/A	N/A	N/A
Fernandez et al. (34)	1996	0 (0)	4 (9)	0 (0)	0 (0)	N/A	N/A	N/A	N/A	0 (0)	0 (0)
Grieder et al. (51)^a^	2010	5 (4.2)	9 (8)	3 (2.5)	9 (8)	N/A	N/A	N/A	N/A	N/A	N/A
Huang et al. (52)	2021	**↓4** **(4.7)**	**↑28** **(19.4)**	N/A	N/A	12 (14)	28 (20)	**↓12.11 ± 3.86**	**↑15.51 ± 9.54**	0 (0)	2 (1.4)
Huh et al. (36)	2010	6 (5.8)	13 (11)	N/A	N/A	N/A	N/A	9.46 ± 2.37	9.81 ± 3.03	N/A	N/A
Kim et al. (53)	2013	**↓17** **(4.1)**	**↑59** **(8)**	0 (0)	7 (1)	N/A	N/A	N/A	N/A	N/A	N/A
Liu et al. (37)	2003	**↓0** **(0)**	**↑8** **(2.2)**	**↓3 (2.5)**	**↑12 (3.3)**	N/A	N/A	N/A	N/A	N/A	N/A
Oliver et al. (55)	2012	**↓7** **(13.5)**	**↑15** **(28.9)**	N/A	N/A	N/A	N/A	N/A	N/A	3 (5.8)	4 (7.7)
Saldaña-Cortés et al. (38)	2009	4 (28.6)	12 (50)	N/A	N/A	N/A	N/A	12.6 ± 2.6	12.9 ± 2.6	1 (7.1)	1 (4.1)
Sdralis et al. (56)	2019	5 (14.3)	3 (13.7)	N/A	N/A	N/A	N/A	N/A	N/A	N/A	N/A
Sieda et al. (57)	2015	3 (8.6)	7 (20)	N/A	N/A	N/A	N/A	5 ± 1.7	7 ± 2.3	N/A	N/A
Silecchia et al. (39)	2006	0 (0)	2 (1.8)	**↓0 (0)**	**↑8 (7.2)**	N/A	N/A	7.0 ± 1.6	7.0 ± 1.8	0 (0)	0 (0)
Upadhyaya et al. (40)	2007	**↓2** **(9.1)**	**↑10** **(43.5)**	N/A	N/A	N/A	N/A	N/A	N/A	2 (9.1)	6 (26)

*N/A, Not available;* ↓, *significantly lower;* ↑, *significantly higher.*

*I*, *Intervention group (coated or reinforced anastomoses); and C*, *control group***.**

*^a^* *Abstract.*

*^b^* *If given in “median (interquaratile range)” or “median (range: minimum - maximum”, values were converted using the Box-Cox (BC) method of McGrath et al. 2020 (47) to estimate the sample mean and standard deviation.* *The bold indicates significant outcomes.*

### Risk of Bias Assessment

The quality of the included studies was assessed by two investigators (K.C. and F.S.) independently. Systematic assessment of the risk of bias for randomized controlled studies and nonrandomized studies of interventions was conducted using the Risk of Bias 2 (RoB 2) tool (44) and the Risk Of Bias In Nonrandomized Studies of Interventions (ROBINS-I) tool (45), according to the recommendations in the Cochrane Handbook for Reviews of Interventions (41). The Newcastle–Ottawa Scale (NOS) for cohort studies (46), a commonly used and established tool, was used to evaluate the quality of included observational studies. We defined any study with an NOS score of >7 as high quality, 5–7 as moderate, and <5 as low quality. Any disagreements were resolved in consensus with the third investigator (S.R.).

### Synthesis Method

All statistical analyses in this review were carried out using Review Manager software version 5.3. (Nordic Cochrane Centre, Copenhagen, Denmark) and the JASP Team (2021; JASP, version 0.16). Results with a *p*-value of <0.05 are considered significant. Values given in the unit “median (interquaratile range)” or “median (range: minimum - maximum)” were converted using the Box-Cox method of McGrath et al. (48) to estimate the sample mean and standard deviation. Heterogeneity across studies was analyzed using the statistical *I*^2^ test, considering *I*^2 ^≥ 50% as substantial heterogeneity (48). In case of substantial heterogeneity (*I*^2 ^≥ 50%), the random-effects model was used to conduct the meta-analyses; for *I*^2 ^< 50%, the fixed-effects model was utilized.

Potential publication bias was examined using Egger’s test (49) for funnel plot asymmetry for outcomes including ≥10 studies, as it is not recommended to conduct the test in the case of fewer studies included (41). To evaluate the stability of our outcomes, we conducted a sensitivity analysis by evaluating the impact of excluding one study at a time on the pooled OR, regardless of the observed heterogeneity. Subgroup analyses were planned *a priori* to assess potential risk factors on studied postoperative outcomes and patient groups at higher risk for complications. The predefined subgroups, assessed in secondary analysis, were stratified by study design, intervention used (collagen-based laminar biomaterials and/or fibrin sealants), age group (adult or pediatric), location of anastomoses (esophagus, stomach, small intestine, colon, and/or rectum), and indication for surgery. Differences in the outcomes across these subgroups were assessed and reported using the test for subgroup differences (TSD).

In subject to the calculated *I*^2^ percentage, either the random-effects model (*I*^2 ^≥ 50%) or the fixed-effects model (*I*^2 ^< 50%) was used to summarize and depict pooled ORs with 95% CIs in a forest plot.

## Results

**Figure 1:**
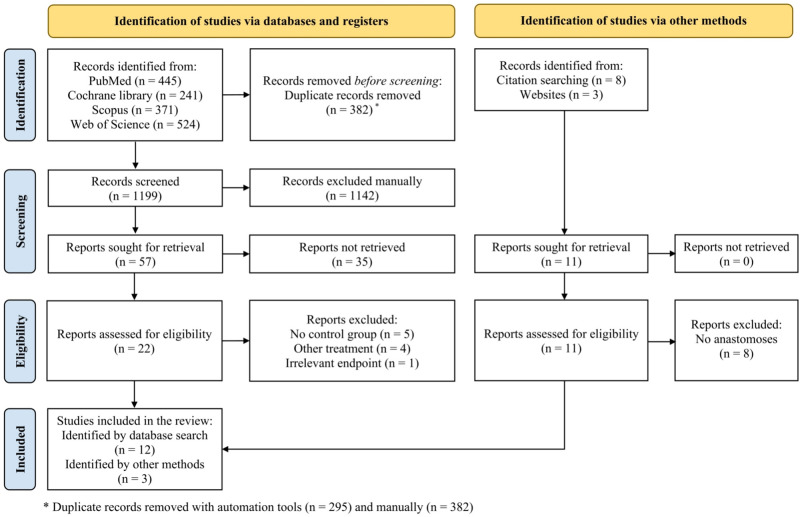
Study flow diagram according to the Preferred Reporting Items for Systematic Review and Meta-Analyses (PRISMA) Statement 2020 (42).

In summary, we identified 1,581 studies through electronic database search and 11 studies through citation and website search, out of which 382 duplicates were removed. Title and abstracts of 1,199 studies were screened manually, and 1,142 studies lacking eligibility were excluded. Of 57 eligible studies thus-acquired for full-text analysis, 35 could not be retrieved, leaving 22 studies originating from the database search and 11 studies identified by other methods. After full-text analysis, 10 of 22 studies were excluded: five studies without a control group, four studies using other interventions, and one study with an irrelevant endpoint. Of the 11 studies identified through citation and website search, eight studies without the formation of an anastomosis were excluded. Finally, 15 studies (34, 36–40, 50–58) were analyzed quantitatively and qualitatively for this systematic review and included in our meta-analyses (**Figure 1**).

### Study Characteristics

This systematic review and meta-analysis evaluates five RCTs (34, 39, 40, 55, 56), three nonrandomized intervention studies (NRSs) (37, 38, 58), four retrospective cohort studies (RCSs) (50, 52–54), two prospective cohort studies (PCS) (36, 57), and one abstract (51). These studies were published between 1996 and 2021 and were conducted in China (52), Egypt (57), France (50), Greece (56), India (40), Italy (39, 54), Korea (36, 53), Mexico (38), Spain (34, 55, 58), Switzerland (51), and the USA (37).

Of 3,630 patients included in 15 studies, 1,387 patients received an intervention, while 2,243 served as a control. To cover the anastomoses, collagen-based laminar biomaterials were utilized in 252 patients (50, 54, 58), and fibrin sealants were utilized in 1,135 cases (34, 37–40, 51–53, 55–57). The majority of studies examined adult patients (34, 36, 37, 39, 50–58) undergoing intestinal surgery for malignant tumors (34, 36, 50–54, 56–58), benign lesions (such as diverticulitis, inflammatory bowel disease, or any kind of nonmalignant intestinal obstruction) (50, 55, 57), or bariatric surgery due to morbid obesity (37, 39). Pediatric patients were examined in two studies (38, 40); indications for surgery were either congenital esophageal atresia with tracheoesophageal fistula (40) or caustic esophageal injury (38) (**Table 1**).

In all cases, regardless of the anastomotic location or technique, intestinal anastomoses of patients in the intervention group were either reinforced or covered externally with either collagen-based laminar biomaterials (Collatamp or TachoSil) (50, 54, 58) or fibrin sealants (Tisseel, Tissucol, Greenplast, Bioseal or Quixil) (34, 36–40, 51–53, 55–57). Patients in the control group received the same surgical procedure as the intervention group but without covering the anastomoses with any substance. Detailed surgical characteristics, including surgical intervention and anastomotic technique, are depicted in **Table 2**.

Postoperative AL was assessed in 14 studies (34, 36–40, 51–58), out of which five (37, 40, 52, 53, 55) found a significantly lower AL rate within the intervention group. Reoperation and C-DMC rates were found to occur significantly less common in patients with sealed anastomoses in two (37, 39) out of six and one (50) out of two studies, respectively. Two out of six studies (52, 54) reported significantly longer hospitalizations for patients in the control group. Differences between the study groups in regard to mortality rates could not be detected in seven studies (34, 38–40, 52, 54, 55) (**Table 3**).

### Risk of Bias Assessment

Risk of bias assessment was performed for all but one study (51), representing an abstract instead of a full-text article (**Supplementary Table S2**).

To assess the risk of bias for included RCTs (34, 39, 40, 55, 56), the RoB 2 tool (44) was utilized, and for nonrandomized studies (37, 38, 58), the ROBINS-I tool (45) was applied, according to the recommendations in the *Cochrane Handbook for Reviews of Interventions* (41). All of these studies presented either some concerns (RCT) (34, 39, 40, 55, 56) or moderate risk of bias (NRS) (37, 38, 58).

The NOS for cohort studies (46) was used to assess the quality of the six included observational studies (36, 50, 52–54, 57). The risk of bias based on this quality assessment presented the majority of studies (36, 50, 52–54) as being of moderate quality (*n* = 5; NOS score 6–7), while one study (57) appeared to be low in quality (NOS < 5).

### Result of Synthesis

#### Postoperative Anastomotic Leakage Rates

**Figure 2:**
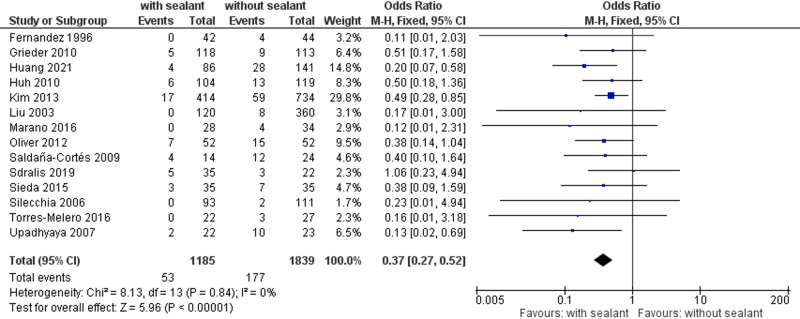
Fixed-effects meta-analysis for the postoperative anastomotic leakage rate in the intervention (coated or reinforced anastomoses) and control group. The forest plot of all studies is included.

Overall, 14 studies (34, 36–40, 51–58) reported postoperative AL rates occurring in 53 (4.5%) of 1,185 patients in the intervention group and 177 (9.6%) of 1,839 patients in the control group. The AL rate was significantly lower for patients with coated anastomoses using fixed-effects meta-analysis (OR, 0.37; 95% CI, 0.27–0.52; *p *< 0.00001) (**Figure 2**).

Studies were homogeneous (*I*^2^ = 0%; *p* = 0.84), and no publication bias was observed (Egger’s test *p* = 0.227). Observed results remained stable throughout sensitivity analyses, excluding one study at a time (**Table 4**).

**Table 4:** Fixed-effects meta-analysis for postoperative anastomotic leakage in the intervention and control group.

Postoperative anastomotic leakage	Odds ratio (OR): fixed-effects model	Heterogeneity	Eggers test
Overall	OR, 0.37; 95% CI, 0.27–0.52; ***p *< 0.00001 [↓(I); ↑(C)]**	*I*^2^ = 0%; *p *= 0.84	*p *= 0.227
**Sensitivity analyses**			
**Excluded study**	**OR: fixed-effects model**	**Heterogeneity**	
Fernandez et al. (34)	OR, 0.38; 95% CI, 0.28–0.53; ***p *< 0.00001 [↓(I); ↑(C)]**	*I*^2^ = 0%; *p *= 0.83	
Grieder et al. (51)	OR, 0.36; 95% CI, 0.26–0.51; ***p *< 0.00001 [↓(I); ↑(C)]**	*I*^2^ = 0%; *p *= 0.79	
Huang et al. (52)	OR, 0.40; 95% CI, 0.29–0.57; ***p *< 0.00001 [↓(I); ↑(C)]**	*I*^2^ = 0%; *p *= 0.90	
Huh et al. (36)	OR, 0.36; 95% CI, 0.26–0.51; ***p *< 0.00001 [↓(I); ↑(C)]**	*I*^2^ = 0%; *p *= 0.79	
Kim et al. (53)	OR, 0.33; 95% CI, 0.22–0.48; ***p *< 0.00001 [↓(I); ↑(C)]**	*I*^2^ = 0%; *p *= 0.84	
Liu et al. (37)	OR, 0.38; 95% CI, 0.27–0.53; ***p *< 0.00001 [↓(I); ↑(C)]**	*I*^2^ = 0%; *p *= 0.80	
Marano et al. (54)	OR, 0.38; 95% CI, 0.28–0.53; ***p *< 0.00001 [↓(I); ↑(C)]**	*I*^2^ = 0%; *p *= 0.82	
Oliver et al. (55)	OR, 0.37; 95% CI, 0.27–0.53; ***p *< 0.00001 [↓(I); ↑(C)]**	*I*^2^ = 0%; *p *= 0.77	
Saldaña-Cortés et al. (38)	OR, 0.37; 95% CI, 0.27–0.52; ***p *< 0.00001 [↓(I); ↑(C)]**	*I*^2^ = 0%; *p *= 0.77	
Sdralis et al. (56)	OR, 0.36; 95% CI, 0.26–0.50; ***p *< 0.00001 [↓(I); ↑(C)]**	*I*^2^ = 0%; *p *= 0.89	
Sieda et al. (57)	OR, 0.37; 95% CI, 0.27–0.52; ***p *< 0.00001 [↓(I); ↑(C)]**	*I*^2^ = 0%; *p *= 0.78	
Silecchia et al. (39)	OR, 0.38; 95% CI, 0.27–0.52; ***p *< 0.00001 [↓(I); ↑(C)]**	*I*^2^ = 0%; *p *= 0.78	
Torres-Melero et al. (58)	OR, 0.37; 95% CI, 0.27–0.53; ***p *< 0.00001 [↓(I); ↑(C)]**	*I*^2^ = 0%; *p *= 0.80	
Upadhyaya et al. (40)	OR, 0.39; 95% CI, 0.28–0.54; ***p *< 0.00001 [↓(I); ↑(C)]**	*I*^2^ = 0%; *p *= 0.90	

↓, *Significantly lower;* ↑, *significantly higher; I, intervention group (coated or reinforced anastomoses); C, control group.* *The bold indicates significant outcomes.*

Subgroup analyses found no subgroup differences for subgroups stratified by study design (TSD: *p* = 0.74), intervention used (TSD: *p* = 0.33), age group (TSD: *p* = 0.40), anastomotic location (TSD: *p* = 0.63), indication for surgery (TSD: *p* = 0.66), and its subclassification (TSD: *p* = 0.45) (**Table 5**).

**Table 5:** Subgroup analyses of fixed-effects meta-analysis for postoperative anastomotic leakage.

Subgroup analyses		
Subgroup	Odds ratio (OR): fixed-effects model	Test for subgroup difference
Study design		*p *= 0.75
RCT	OR, 0.33; 95% CI, 0.17–0.65; ***p *= 0.001 [↓(I); ↑(C)]**	
NRS	OR, 0.27; 95% CI, 0.09–0.87; ***p *= 0.03 [↓(I); ↑(C)]**	
OS	OR, 0.40; 95% CI, 0.27–0.60; ***p *< 0.00001 [↓(I); ↑(C)]**	
Covering		*p *= 0.33
C-BLB	OR, 0.13; 95% CI, 0.02–1.12; *p = *0.06	
FS	OR, 0.39; 95% CI, 0.28–0.54; ***p *< 0.00001 [↓(I); ↑(C)]**	
Age group		*p *= 0.40
Adult	OR, 0.39; 95% CI, 0.28–0.55; ***p *< 0.00001 [↓(I); ↑(C)]**	
Pediatric	OR, 0.24; 95% CI, 0.08–0.69; ***p = *0.008 [↓(I); ↑(C)]**	
Anastomotic location		*p *= 0.63
Esophagus	OR, 0.28; 95% CI, 0.15–0.55; ***p = *0.0002 [↓(I); ↑(C)]**	
Esophagojejunal or gastrojejunal	OR, 0.28; 95% CI, 0.12–0.67; ***p = *0.004 [↓(I); ↑(C)]**	
Gastrojejunal (bariatric surgery)	OR, 0.19; 95% CI, 0.02–1.58; *p = *0.12	
Colorectal	OR, 0.47; 95% CI, 0.31–0.71; ***p = *0.0004 [↓(I); ↑(C)]**	
Miscellaneous	OR, 0.38; 95% CI, 0.28–0.51; *p = *0.06	
Indication for surgery		*p *= 0.66
Malignant tumor	OR, 0.40; 95% CI, 0.28–0.58; ***p****** *< 0.00001 [↓(I); ↑(C)]**	
Obesity (bariatric surgery)	OR, 0.19; 95% CI, 0.02–1.58; *p = *0.12	
Miscellaneous	OR, 0.31; 95% CI, 0.15–0.63; ***p = *0.001 [↓(I); ↑(C)]**	
Indication for surgery (subclassified)	* *	*p *= 0.45
Upper GIT malignancy	OR, 0.26; 95% CI, 0.12–0.56; ***p****** *= 0.0005 [↓(I); ↑(C)]**	
Lower GIT malignancy	OR, 0.47; 95% CI, 0.31–0.71; ***p****** *= 0.0004 [↓(I); ↑(C)]**	
Obesity (bariatric surgery)	OR, 0.19; 95% CI, 0.02–1.58; *p = *0.12	
Miscellaneous	OR, 0.31; 95% CI, 0.15–0.63; ***p = *0.001 [↓(I); ↑(C)]**	

↓, *Significantly lower;* ↑, *significantly higher; I, intervention group (coated or reinforced anastomoses); C, control group; RCT, randomized controlled trial; NRS, nonrandomized study; OS, observational study; C-BLB, collagen-based laminar biomaterial; FS, fibrin sealant; GIT, gastrointestinal tract*. *The bold indicates significant outcomes.*

#### Postoperative Reoperation Rates

**Figure 3:**
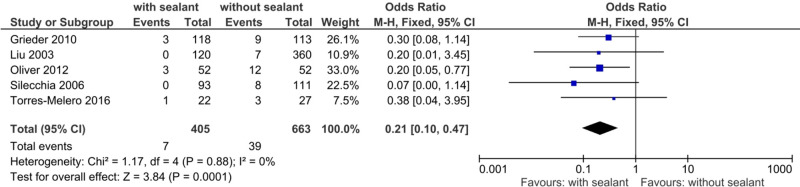
Fixed-effects meta-analysis for the postoperative reoperation rate in the intervention (coated or reinforced anastomoses) and control group. The forest plot of all studies is included.

A total of five studies (37, 39, 51, 55, 58) examined the postoperative reoperation rates, occurring in seven (1.7%) of 405 patients in the intervention group and 39 (5.9%) of 663 patients in the control group. Rates of reoperation presented to be significantly lower for patients in the intervention group using fixed-effects meta-analysis (OR, 0.21; 95% CI, 0.10–0.47; *p *= 0.0001) (**Figure 3**).

Studies were homogeneous (*I*^2^ = 0%; *p* = 0.88), and results remained stable in sensitivity analyses. Subgroup analyses found no subgroup differences for subgroups stratified by study design (TSD: *p* = 0.71), intervention used (TSD: *p* = 0.60), anastomotic location (TSD: *p* = 0.64), and indication for surgery (TSD: *p* = 0.64) (**Table 6**).

**Table 6:** Fixed-effects meta-analysis for postoperative reoperation in the intervention and control group.

Postoperative reoperation	Odds ratio (OR): fixed-effects model	Heterogeneity
Overall	OR, 0.21; 95% CI, 0.10–0.47; ***p *= 0.0001 [↓(I); ↑(C)]**	*I*^2^ = 0%; *p *= 0.88
**Sensitivity analyses**		
**Excluded study**	**OR: fixed-effects model**	**Heterogeneity**
Grieder et al. (51)	OR, 0.18; 95% CI, 0.07–0.48; ***p *= 0.0007 [↓(I); ↑(C)]**	*I*^2^ = 0%; *p *= 0.82
Liu et al. (37)	OR, 0.21; 95% CI, 0.09–0.48; ***p *= 0.0002 [↓(I); ↑(C)]**	*I*^2^ = 0%; *p *= 0.76
Oliver et al. (55)	OR, 0.21; 95% CI, 0.08–0.57; ***p *= 0.002 [↓(I); ↑(C)]**	*I*^2^ = 0%; *p *= 0.76
Silecchia et al. (39)	OR, 0.25; 95% CI, 0.11–0.58; ***p *= 0.001 [↓(I); ↑(C)]**	*I*^2^ = 0%; *p *= 0.96
Torres-Melero et al. (58)	OR, 0.20; 95% CI, 0.08–0.46; ***p *= 0.0002 [↓(I); ↑(C)]**	*I*^2^ = 0%; *p *= 0.81
**Subgroup analyses**		
**Subgroup**	**OR: fixed-effects model**	**Test for subgroup difference**
Study design		*p *= 0.71
RCT	OR, 0.15; 95% CI, 0.04–0.49; ***p *= 0.002 [↓(I); ↑(C)]**	
NRS	OR, 0.27; 95% CI, 0.04–1.65; *p *= 0.16	
OS	OR, 0.30; 95% CI, 0.08–1.14; *p *= 0.08	
Covering		*p *= 0.60
C-BLB	OR, 0.38; 95% CI, 0.04–3.95; *p = *0.42	
FS	OR, 0.20; 95% CI, 0.08–0.46; ***p *= 0.0002 [↓(I); ↑(C)]**	
**Age group (adults only)**		
Anastomotic location		*p *= 0.64
Gastrojejunal (bariatric surgery)	OR, 0.11; 95% CI, 0.01–0.81; ***p = *0.03 [↓(I); ↑(C)]**	
Colorectal	OR, 0.32; 95% CI, 0.10–1.02; ***p = *0.05 [↓(I); ↑(C)]**	
Miscellaneous	OR, 0.20; 95% CI, 0.05–0.77; ***p = *0.02 [↓(I); ↑(C)]**	
Indication for surgery		*p *= 0.64
Malignant tumor (lower GIT)	OR, 0.32; 95% CI, 0.10–1.02; ***p****** *= 0.05 [↓(I); ↑(C)]**	
Obesity (bariatric surgery)	OR, 0.11; 95% CI, 0.01–0.81; ***p = *0.03 [↓(I); ↑(C)]**	
Miscellaneous	OR, 0.20; 95% CI, 0.05–0.77; ***p = *0.02 [↓(I); ↑(C)]**	

↓, *Significantly lower;* ↑, *significantly higher; I, intervention group (coated or reinforced anastomoses); C, control group; RCT, randomized controlled trial; NRS, nonrandomized study; OS, observational study; C-BLB, collagen-based laminar biomaterial; FS, fibrin sealant; GIT, gastrointestinal tract.* *The bold indicates significant outcomes.*

#### Overall Postoperative Clavien–Dindo Major Complication Rates

**Figure 4:**
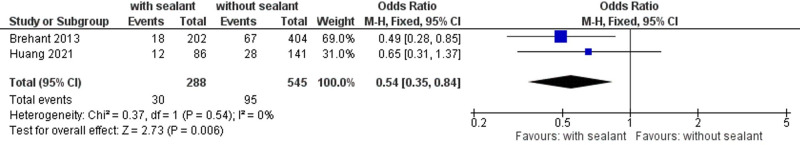
Fixed-effects meta-analysis for the postoperative major complication rate according to the Clavien–Dindo classification of surgical complications (43) in the intervention (coated or reinforced anastomoses) and control group. The forest plot of all studies is included.

Two studies (50, 52) evaluated the incidence of postoperative major complications according to the Clavien–Dindo classification of surgical complications (43). In total, 30 (10.4%) of 288 patients with external anastomotic coating and 95 (17.4%) of 545 patients in the control group developed postoperative C-DMC. The intervention group presented with significantly lower C-DMC rates using fixed-effects meta-analysis (OR, 0.54; 95% CI, 0.35–0.84; *p *= 0.006). Studies were homogeneous (*I*^2^ = 0%; *p* = 0.54) (**Figure 4**).

#### Length of Hospitalization

**Figure 5:**
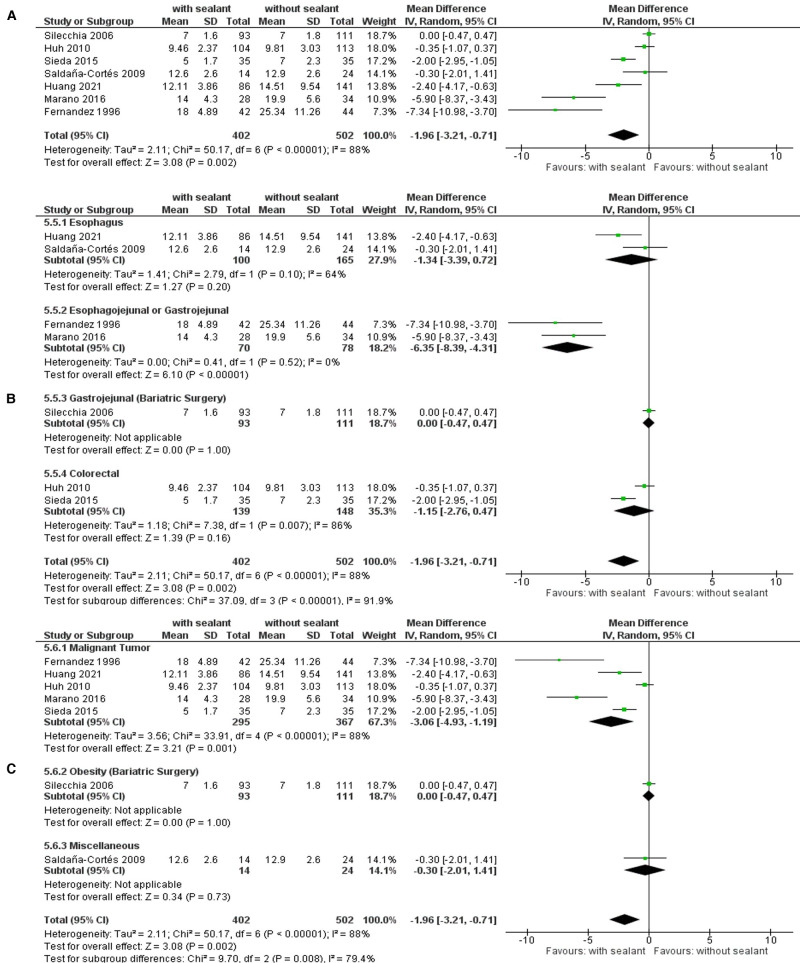
Random-effects meta-analysis for the length of hospitalization in the intervention (coated or reinforced anastomoses) and control group. (**A**) Forest plot of all studies included. (**B**) Forest plot of subgroup analysis stratified by location of anastomoses. (**C**) Forest plot of subgroup analysis stratified by indication of surgery.

Another seven studies (34, 36, 38, 39, 52, 54, 57) monitored the length of hospitalization. The overall length of hospitalization was significantly shorter for patients in the intervention group compared to those for patients in the control group using the random-effects model meta-analysis to calculate the weighted mean difference (WMD, −1.96; 95% CI: −3.21, −0.71; *p *= 0.002). Studies showed significant substantial heterogeneity (*I*^2^ = 88%; *p* < 0.00001) but remained stable throughout sensitivity analyses. Subgroup analyses found a significant subgroup difference when the patients were stratified according to the intervention used (TSD: *p *= 0.0010), anastomotic location (TSD: *p *< 0.00001), indication for surgery (TSD: *p *= 0.001), and its subclassification (TSD: *p *= 0.001) (**Figure 5**).

Patients in the intervention group presented with a significantly shorter time of hospitalization compared to the control group if undergoing intestinal surgical procedures for malignant gastrointestinal tumors (WMD, −3.06; 95% CI: −4.93, −1.19; *p *= 0.001), especially if they were located in the upper GIT (WMD, −4.94; 95% CI: −7.98, −1.90; *p *= 0.001) and were operated with the creation of an esophagojejunal or gastrojejunal anastomosis (WMD, −2.28; 95% CI: −6.35, −4.31; *p *< 0.00001) (**Table 7**).

**Table 7:** Random-effects meta-analysis for the length of hospitalization in the intervention and control group.

Length of hospitalization	Weighted mean difference (WMD): random-effects model	Heterogeneity
Overall	WMD, −1.96; 95% CI: −3.21, −0.71; ***p *= 0.002 [↓(I); ↑(C)]**	*I*^2^ = 88%; ***p *< 0.00001**
**Sensitivity analyses**		
**Excluded study**	**WMD: random-effects model**	**Heterogeneity**
Fernandez et al. (34)	WMD, −1.48; 95% CI: −2.62, −0.33; ***p = *0.01 [↓(I); ↑(C)]**	*I*^2^ = 86%; ***p *< 0.00001**
Huang et al. (52)	WMD, −1.90; 95% CI: −3.24, −0.55; ***p = *0.006 [↓(I); ↑(C)]**	*I*^2^ = 89%; ***p *< 0.00001**
Huh et al. (36)	WMD, −2.50; 95% CI: −4.21, −0.79; ***p = *0.004 [↓(I); ↑(C)]**	*I*^2^ = 90%; ***p *< 0.00001**
Marano et al. (54)	WMD, −1.36; 95% CI: −2.46, −0.25; ***p = *0.02 [↓(I); ↑(C)]**	*I*^2^ = 84%; ***p *< 0.00001**
Saldaña-Cortés et al. (38)	WMD, −2.28; 95% CI: −3.68, −0.87; ***p = *0.001 [↓(I); ↑(C)]**	*I*^2^ = 90%; ***p *< 0.00001**
Sieda et al. (57)	WMD, −1.99; 95% CI: −3.41, −0.57; ***p = *0.006 [↓(I); ↑(C)]**	*I*^2^ = 88%; ***p *< 0.00001**
Silecchia et al. (39)	WMD, −2.53; 95% CI: −4.12, −0.94; ***p = *0.002 [↓(I); ↑(C)]**	*I*^2^ = 86%; ***p *< 0.00001**
**Subgroup analyses**		
**Subgroup**	**WMD: random-effects model**	**Test for subgroup difference**
Study design		*p *= 0.22
RCT	WMD, −3.44; 95% CI: −10.62, 3.74; *p = *0.35	
NRS	WMD, −0.30; 95% CI: −2.01, 1.41; *p = *0.73	
OS	WMD, −2.36; 95% CI: −4.10, −0.61; ***p = *0.008 [↓(I); ↑(C)]**	
Covering		***p *= 0.0010**
C-BLB	WMD, −5.90; 95% CI: −8.37, −3.43; ***p *< 0.00001 [↓(I); ↑(C)]**	
FS	WMD, −1.36; 95% CI: −2.46, −0.25; ***p = *0.02 [↓(I); ↑(C)]**	
Age group		*p *= 0.08
Adult	WMD, −2.28; 95% CI: −3.68, −0.87; ***p = *0.001 [↓(I); ↑(C)]**	
Pediatric	WMD, −0.30; 95% CI: −2.01, 1.41; *p = *0.73	
Anastomotic location		***p *< 0.00001**
Esophagus	WMD, −1.34; 95% CI: −3.39, 0.72; *p = *0.2	
Esophagojejunal or gastrojejunal	WMD, −2.28; 95% CI: −6.35, −4.31; ***p *< 0.00001 [↓(I); ↑(C)]**	
Gastrojejunal (bariatric surgery)	WMD, 0.0; 95% CI: −0.47, 0.47; *p = *1.0	
Colorectal	WMD, −1.15; 95% CI: −2.76, 0.47; *p = *0.16	
Indication for surgery		***p *= 0.008**
Malignant tumor	WMD, −3.06; 95% CI: −4.93, −1.19; ***p *= 0.001 [↓(I); ↑(C)]**	
Obesity (bariatric surgery)	WMD, 0.0; 95% CI: −0.47, 0.47; *p = *1.0	
Miscellaneous	WMD, −0.30; 95% CI: −2.01, 1.41; *p = *0.73	
Indication for surgery (subclassified)	* *	***p *= 0.010**
Upper GIT malignancy	WMD, −4.94; 95% CI: −7.98, −1.90; ***p *= 0.001 [↓(I); ↑(C)]**	
Lower GIT malignancy	WMD, −1.15; 95% CI: −2.76, 0.47; *p = *0.16	
Obesity (bariatric surgery)	WMD, 0.0; 95% CI: −0.47, 0.47; *p = *1.0	
Miscellaneous	WMD, −0.30; 95% CI: −2.01, 1.41; *p = *0.73	

↓, *Significantly lower;* ↑, *significantly higher; I, intervention group (coated or reinforced anastomoses); C, control group; RCT, randomized controlled trial; NRS, nonrandomized study; OS, observational study; C-BLB, collagen-based laminar biomaterial; FS, fibrin sealant; GIT, gastrointestinal tract.* *The bold indicates significant outcomes.*

#### Postoperative Mortality Rate

**Figure 6:**
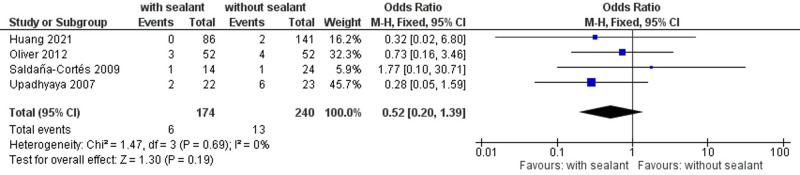
Fixed-effects meta-analysis for the postoperative mortality rate in the intervention (coated or reinforced anastomoses) and control group. The forest plot of all studies is included.

In total, four studies recorded postoperative mortality rates (38, 40, 52, 55), occurring in six (3.4%) of 174 patients with fibrin sealant-coated anastomoses and 13 (5.5%) of 240 patients in the control group. No significant differences were found between the studied groups using fixed-effects meta-analysis (OR, 0.52; 95% CI, 0.20–1.39; *p = *0.19) (**Figure 6**).

Studies were homogeneous (*I*^2^ = 0%; *p = *0.69) and remained stable in sensitivity analyses. Subgroup analyses found no significant subgroup difference for subgroups stratified by study design (TSD: *p* = 0.66), age group (TSD: *p* = 0.78), anastomotic location (TSD: *p* = 0.59), and indication for surgery (TSD: *p* = 0.74) (**Table 8**).

**Table 8:** Fixed-effects meta-analysis for postoperative mortality in the intervention and control goup.

Mortality	Odds ratio (OR): fixed-effects model	Heterogeneity
Overall	OR, 0.52; 95% CI, 0.20–1.39; *p = *0.19	*I*^2^ = 0%; *p = *0.69
**Sensitivity analyses**		
**Excluded study**	**OR: fixed-effects model**	**Heterogeneity**
Huang et al. (52)	OR, 0.56; 95% CI, 0.20–1.59; *p = *0.28	*I*^2^ = 0%; *p = *0.51
Oliver et al. (55)	OR, 0.42; 95% CI, 0.12–1.52; *p = *0.19	*I*^2^ = 0%; *p = *0.55
Saldaña-Cortés et al. (38)	OR, 0.44; 95% CI, 0.15–1.28; *p = *0.13	*I*^2^ = 0%; *p = *0.70
Upadhyaya et al. (40)	OR, 0.72; 95% CI, 0.22–2.42; *p = *0.60	*I*^2^ = 0%; *p = *0.72
**Subgroup analyses**		
**Subgroup**	**OR: Fixed-effects model**	**Test for subgroup difference**
Study design		*p *= 0.66
RCT	OR, 0.47; 95% CI, 0.15–1.46; *p = *0.19	
NRS	OR, 1.77; 95% CI, 0.10–30.71; *p = *0.70	
OS	OR, 0.32; 95% CI, 0.02–6.80; *p = *0.47	
Covering (FS only)		
Age group		*p *= 0.78
** ** Adult	OR, 0.60; 95% CI, 0.15–2.31; *p = *0.46	
** ** Pediatric	OR, 0.45; 95% CI, 0.11–1.87; *p = *0.27	
** **Anastomotic Location		*p *= 0.59
** ** Esophagus	OR, 0.42; 95% CI, 0.12–1.52; *p = *0.19	
** ** Miscellaneous	OR, 0.73; 95% CI, 0.16–3.46; *p = *0.70	
** **Indication for Surgery		*p *= 0.74
** **Malignant Tumor (upper GIT)	OR, 0.32; 95% CI, 0.02–6.8; *p = *0.47	
** ** Miscellaneous	OR, 0.56; 95% CI, 0.20–1.59; *p = *0.28	

*RCT, randomized controlled trial; NRS, nonrandomized study; OS, observational study; FS, fibrin sealant; GIT, gastrointestinal tract.*

## Discussion

This systematic review and meta-analysis gives an overview of the efficacy of externally covering anastomoses with collagen-based laminar biomaterials or fibrin sealants in reducing postoperative rates of AL and its accompanying sequelae for patients undergoing surgery with the formation of an intestinal anastomosis.

The meta-analyses found significant differences for postoperative AL (**Figure 2**), reoperation rates (**Figure 3**), C-DMC (43) (**Figure 4**), and length of hospitalization (**Figure 5**). However, no significant differences between the studied groups were found in the postoperative mortality rate, even after conducting sensitivity and subgroup analyses (**Figure 6** and **Table 8**).

A significant decrease in AL (**Figure 2**; **Tables 4** and **5**) and reoperation rate (**Figure 3**; **Table 6**) was found for patients with intestinal anastomoses covered either by collagen-based laminar biomaterials or by fibrin sealants. Sensitivity analyses confirmed the stability of these results. Subgroup analyses did not find any difference between the collagen-based laminar biomaterials and fibrin sealants in regard to their protective action. Furthermore, the outcomes remained significant regardless of the study design, age group studied, location of anastomoses, or indication of surgery. Postoperative major complications, according to the Clavien–Dindo classification for surgical complications (43), were shown to be significantly lower in the intervention group than those in the control group. Since only two studies (50, 52) reported complications categorized by this classification, no sensitivity or subgroup analysis could be conducted (**Figure 4**).

The length of hospitalization appeared to be significantly shorter for patients in the intervention group (**Figure 5**). These results remained stable throughout sensitivity analyses, and subgroup analyses did not find differences between subgroups stratified by study design, intervention used, or age group. However, a significant subgroup difference was observed for subgroups stratified by the location of anastomoses and the indication for surgery. In comparison to the control group, patients in the intervention group presented with a significantly shorter time of hospitalization if undergoing intestinal surgery with esophagojejunal or gastrojejunal anastomoses or if the indication for surgery was a malignant tumor, especially the case with upper gastrointestinal malignancies (**Table 7**).

No difference between the intervention and control group could be found in regard to postoperative mortality rates, even after performing sensitivity and subgroup analyses (**Figure 6**; **Table 8**). This outcome should be interpreted with caution, as not all studies reporting AL also reported postoperative mortality rates. To evaluate the effect of coating intestinal anastomoses with collagen-based laminar biomaterials or fibrin sealants on postoperative mortality rates, future studies should allow a longer follow-up for their patients to ensure postoperative mortality is not missed.

On the downside of the ambiguous outcomes presented in different experimental animal studies (22–31, 33, 59, 60), fibrin sealants have been utilized already in human trials, showing positive effects. Sealing postoperatively occurring anastomotic leaks of the upper and lower GIT with fibrin sealants endoscopically has been conducted with successful therapeutic outcomes (61–63). Endoscopic applications have shown to reduce exudation from the leakage site, systemic inflammatory response, and clinical symptoms of treated patients (61) and seem to serve as an efficient and safe option to manage postoperative ALs (62).

Furthermore, a recently published systematic review reported mainly positive effects on AL prevention and treatment upon covering esophageal anastomoses with collagen-based laminar biomaterials or fibrin sealants (64). Promising effects for staple-line reinforcement with absorbable materials such as fibrin sealants were reported as well for colorectal procedures (65). In the case of bariatric surgical procedures, Chen et al. (66) conducted a meta-analysis of six randomized controlled trials examining the effect of staple-line and anastomotic reinforcement with fibrin sealants on postoperative complications in morbidly obese patients undergoing laparoscopic sleeve gastrectomy or Roux-en-Y-gastric bypass. The authors demonstrated no significant difference between the studied groups’ postoperative AL rates. These results coincide with our findings after conducting a subgroup analysis stratified by indication for surgery. Still, precautions should be taken to compare the results of our subgroup analysis with those of the previously conducted meta-analysis (66), as our study excluded any surgical procedure without the formation of an intestinal anastomosis.

Interestingly, Panda et al. conducted a cost analysis, evaluating the differences in economic burden in regard to resource expenses provided by the healthcare system upon covering colorectal anastomoses with fibrin sealants. The authors concluded that the application of fibrin sealants was not only associated with decreased AL rates but also contributed to cost savings of roughly 22% (using a potential model). These cost savings originate mainly from the reduction in the length of hospitalization due to postoperative reoperations, radiological interventions, and/or transfusions (67). These findings correlate with the observed outcomes of our investigation.

This study showed that coating intestinal anastomoses with collagen-based laminar biomaterials or fibrin sealants resulted in significantly reduced postoperative AL, reoperation, C-DMC rates, and shorter length of hospitalization; nevertheless, there is still room for improvement. A large proportion of postoperative anastomotic leaks is associated with anastomotic infections (68). In a recent study, Anderson et al. (68) investigated cultures of 19 patients with AL and found 74% of these patients' leaks to be colonized with collagenase-producing microorganisms. Furthermore, the authors found the presence of *Enterococcus faecalis* to be significantly associated with the development of AL (68). In the physiology of anastomotic wound healing, the risk of wound failure corresponds to the activity of collagenases (69). As collagen deposition plays a crucial role in adequate anastomotic healing (70), an infection of the anastomosis leads to collagenase enzyme activities exceeding the physiological levels needed for proper wound healing, contributing to anastomotic failure (69, 71). Furthermore, such infections could potentially compromise the functionality of anastomotic coatings with collagen-based laminar biomaterials due to the destructive effect of these microorganisms’ collagenases on the biomaterial’s basic framework. To assure the complete functionality of these adhesive biomaterials and adequate anastomotic healing, infections should be prevented. If sealants would contain both the healing supporting collagen fibrils and antimicrobial substances, effectively protecting the anastomoses and the adhesives from collagenase-producing microorganisms, theoretically, a much higher effect for further reducing postoperative anastomotic complications could be expected.

The results of our analysis have limitations that need to be addressed. The included studies presented with variable study designs and years of publication (1996–2021) and were of moderate quality in most cases. We decided to include studies older than 15 years (34, 37, 39, 40) in our analysis as their interventions are comparable to interventions of studies conducted in the following years and the adhesive biomaterials used correspond to those used in more recent studies. Different types and materials of sealants were compared among patients with different characteristics, such as different age groups and surgical indications, which could have introduced potential biases to our analysis. We addressed this limitation by performing thorough subgroup analyses stratified by these potential confounding factors and investigating the stability of our results by conducting sensitivity analyses, regardless of the observed heterogeneity. Additional sources of potential bias were the possible lack of adequate blinding since none of the five RCTs (34, 39, 40, 55, 56) and three NRSs (37, 38, 58) commented on the outcome assessor’s awareness of intervention, and the potential influence the manufacturer of the adhesive biomaterials used might have had by funding the study. We carefully examined the funding situations with regard to each included study and have come to the conclusion that the manufacturer—to our knowledge—did not present a funding role in any of the included studies nor was an author mentioned to be a representative for the manufacturer. Furthermore, our analysis did not evaluate the effect of coating other types of anastomoses commonly performed in abdominal surgery, such as pancreaticointestinal or biliodigestive anastomoses. Since these types of anastomoses present distinct differences in surgical techniques and specific risks for AL and its associated morbidities, we excluded all types of anastomoses other than intestinal anastomoses of the upper and lower GIT. The risk of biasing the results of our study's observed outcomes would have been potentiated by including these types of anastomoses in our study. Therefore, we did not evaluate these kinds of effects in the present analysis but would recommend analyzing the effects of coating other types of anastomoses commonly performed in abdominal surgery on postoperative complications separately in a further systematic review and meta-analysis in the future.

However, the strength of this study is its uniqueness since this is the first systematic review with a meta-analysis investigating the efficacy of coating intestinal anastomoses with the most commonly utilized absorbable adhesives (20, 21) in reducing postoperative AL rates and its accompanying sequelae.

The outcomes of this systematic review and meta-analysis present some clinical implications and justify the need for future research to consolidate our findings. Furthermore, larger RCTs examining the effects of the studied adhesives in the context of different surgical indications and patient groups need to be conducted. One could ask why coating of intestinal anastomoses with collagen-based laminar biomaterials and/or fibrin sealants has yet not been established in everyday clinical practice. Possible reasons could be the difficult and user-unfriendly application form resulting in additional time expenditure or the low adhesive strength of these biomaterials on intestinal surfaces. Since these adhesive biomaterials have shown significant efficacy in reducing postoperative morbidity after intestinal surgery, future research and innovative developments should address these unfavorable factors.

In conclusion, current evidence suggests that covering intestinal anastomoses with either collagen-based laminar biomaterials or fibrin sealants significantly reduces postoperative rates of AL, reoperation, and C-DMC. Furthermore, with these adhesives, a significant reduction in the length of hospitalization can be observed, especially for patients undergoing surgery for an upper gastrointestinal malignancy. Still, the risk of anastomotic and potential adhesive failure associated with anastomotic infection should be addressed, by investigating the efficacy of antimicrobial collagen-based sealants, for protecting intestinal anastomoses from the deleterious effect of collagenase-producing microorganisms. To consolidate our findings, there is a need for further large RCTs examining the effects of coating intestinal anastomoses with the studied adhesives on postoperative leakage. Aside from that, the effect of coating other types of anastomoses commonly performed in abdominal surgery on postoperative complications should be investigated in future studies. Finally, a simple and user-friendly application form of a somewhat stronger adhesive collagen-based laminar biomaterial and/or fibrin sealant should be developed to establish the possibility of routine use in surgical practice.

## Data Availability

The original contributions presented in the study are included in the article/Supplementary Material further inquiries can be directed to the corresponding author/s.

## Supplementary Material

**Supplementary Table 1 |** Search strategy. Final database search (January 17, 2022).

**Supplementary Table 2 |** Risk of bias assessment for included studies (abstracts excluded).
