# Supplementary material for: Coating of Intestinal Anastomoses for Prevention of Postoperative Leakage: A Systematic Review and Meta-Analysis
Source: Front Surg. 2022 Apr 22;9:882173. doi: 10.3389/fsurg.2022.882173 (PMC9235828; doi:10.3389/fsurg.2022.882173)
Supplement: Supplementary Table 1 | Search strategy. Final database search (January 17, 2022). [file Table1_v1.DOCX]

# Supplementary Table 1. Search Strategy. Final Database Search (January 17, 2022)

| Supplementary Table 1. Search Strategy. Final Database Search (January 17, 2022) | | | | | | | | |
| --- | --- | --- | --- | --- | --- | --- | --- | --- |
|  | **Database** |  |  | **Search Terms** |  |  | **Results, n** |  |
| PubMed (MEDLINE) | | |  | | |  |  |  |
| Search 1: | | | (anastomo* AND (bowel[tiab] OR intestin*[tiab] OR enter*[tiab] OR ile*[tiab] OR colo*[tiab] OR colorect*[tiab] OR rect*[tiab] OR rectal[tiab] OR pancrea*[tiab] OR "intestines"[Mesh] OR "intestine, large" [Mesh] OR "intestine, small") OR "Colon"[Mesh] OR "Ileum"[Mesh] OR "Rectum"[Mesh] OR "Pancreas"[Mesh]) | | | 299677 | | |
| Search 2: | | | ("collagen patch" OR "collagen fleece" OR "collagen mesh" OR "collagen dressing" OR "collagen coating" OR avitene[tiab] OR hemopatch[tiab] OR permacol*[tiab] OR tacho*[tiab] OR "bovine pericard*"[tiab] OR (collag*[tiab] AND matri*[tiab]) "tachosil"[tiab] OR tachocomb[tiab] OR lyostypt[tiab] OR tissuefleece[tiab] OR "collagen sponge"[tiab] OR "fibrin tissue patch"[tiab] OR "collagen matrix"[tiab] OR ("collageneous"[Title/Abstract] AND "matrices"[Title/Abstract]) OR "bovine pericard*"[tiab] OR "fibrin glue"[tiab] OR glue*[tiab] OR "fibrin sealant"[tiab] OR artiss[tiab] OR tisseel[tiab] OR floseal[tiab] OR coseal[tiab] OR beriplast[tiab] OR evicel[tiab] OR "thrombin-JMI"[tiab] OR quixil[tiab] OR vistaseal[tiab] OR dermabond[tiab] OR actifoam[tiab]) | | | 23912 | | |
| Final Search: | | | Search 1 AND Search 2 | | | 445 | | |
|  | | | | | | | | |
| Web of Science | | |  | | | | | |
| Search 1: | | | TS=(anastomo* AND TS=(bowel OR ermacol* OR enter* OR ile* OR colo* OR colorect* OR rect* OR rectal OR permacol*) | | | 31249 | | |
| Search 2: | | | TS= (collagen patch OR collagen fleece OR collagen mesh OR collagen dressing OR collagen coating OR avitene OR hemopatch OR ermacol* OR tacho* OR bovine pericard OR collagen matrix OR collag* matri* OR tachosil OR tachocomb OR lyostypt OR tissuefleece OR collagen sponge OR fibrin tissue patch OR fibrin glue OR glue* OR fibrin sealant OR artiss OR tisseel OR flosea OR coseal OR beriplast OR evicel OR thrombin-JMI OR quixil OR vistaseal OR dermabond OR actifoam) | | | 1319993 | | |
| Final Search: | | | Search 1 AND Search 2 | | | 524 | | |
| Scopus | | |  | | | | | |
| Search 1: | | | TITLE ( ( anastomo* AND bowel ) OR ( anastomo* AND intestin* ) OR ( antastomo* AND enter* ) OR ( anastomo AND ile* ) OR ( anastomo* AND colo* ) OR ( anastomo* AND colorect* ) OR ( anastomo* AND rect* ) OR ( anastomo* AND rectal ) OR ( anastomo* OR pancrea* ) OR ( anastomo* W/2 bowel ) OR ( anastomo* W/2 intestin* ) OR ( antastomo* W/2 enter* ) OR ( anastomo W/2 ile* ) OR ( anastomo* W/2 colo* ) OR ( anastom* W/2 colorect* ) OR ( anastomo* W/2 rect* ) OR ( anastomo* W/2 rectal ) OR ( anastomo* W/2 pancrea* ) ) | | | 251645 | | |
| Search 2: | | | TITLE ( ( ( ( ( ( ( ( ( ( ( ( ( ( ( ( ( ( ( ( ( collagen AND patch ) OR ( collagen AND fleece ) OR ( collagen AND mesh ) OR ( collagen AND dressing ) OR ( collagen AND coating ) OR ( bovine AND pericard* ) OR ( collage* AND matr* ) OR ( collagen AND sponge ) OR ( thrombin AND jmi ) OR ( fibrin AND tissue AND patch ) OR ( fribrin AND glue ) OR ( fibrin AND seal* ) ) OR tachosil ) OR tacho* ) OR avitene ) OR hemopatch ) OR permacol* ) OR tachocomb ) OR lyostypt ) OR tissuefleece ) OR glue* ) OR artiss ) OR tisseel ) OR floseal ) OR coseal ) OR beriplast ) OR evicel ) OR quixil ) OR vistaseal ) OR dermabond ) OR actifoam ) | | | 19483 | | |
| Final Search: | | | Search 1 AND Search 2 | | | 371 | | |
|  | | |  | | |  | | |
| Cochrane Library | | |  | | | | | |
| Search 1: | | | (anastomo*):ti,ab,kw AND (bowel):ti,ab,kw OR (intesin*):ti,ab,kw OR (enter*):ti,ab,kw OR (ile*):ti,ab,kw OR (colo*):ti,ab,kw OR (colorect*):ti,ab,kw OR (rect*):ti,ab,kw | | | 126984 | | |
| Search 2: | | | (collagen patch):ti,ab,kw OR (colalgen fleece):ti,ab,kw OR (collagen mesh):ti,ab,kw OR (collagen dressing):ti,ab,kw OR (collagen coating):ti,ab,kw OR (avitene):ti,ab,kw OR (hemopathc):ti,ab,kw OR (permacol*):ti,ab,kw OR (tacho*):ti,ab,kw OR (bovine pericard*):ti,ab,kw OR (collagen sponge):ti,ab,kw OR (collagen matrix):ti,ab,kw OR (collag* AND matri*):ti,ab,kw OR (tachosil):ti,ab,kw OR (tachocomb):ti,ab,kw OR (lyostypt):ti,ab,kw OR (tissuefleece):ti,ab,kw OR (fibrin tissue patch):ti,ab,kw OR (fibrin glue):ti,ab,kw OR (fibrin sealant):ti,ab,kw OR (artiss):ti,ab,kw OR (tisseel):ti,ab,kw OR (floseal):ti,ab,kw OR (coseal):ti,ab,kw OR (beriplast):ti,ab,kw OR (evicel):ti,ab,kw OR (thrombin JMI):ti,ab,kw OR (quixil):ti,ab,kw OR (vistaseal):ti,ab,kw OR (dermabond):ti,ab,kw OR (actifoam):ti,ab,kw | | | 3244 | | |
| Final Search: | | | Search 1 AND Search 2 | | | 241 | | |
| Additional Data Sets Identified | | |  | | |  | | |
| Citation searching | | |  | | | 8 | | |
| Website | | |  | | | 3 | | |
