# Supplementary material for: Coating of Intestinal Anastomoses for Prevention of Postoperative Leakage: A Systematic Review and Meta-Analysis
Source: Front Surg. 2022 Apr 22;9:882173. doi: 10.3389/fsurg.2022.882173 (PMC9235828; doi:10.3389/fsurg.2022.882173)
Supplement: Supplementary Table 2 | Risk of bias assessment for included studies (abstracts excluded). [file Table2_v1.DOCX]

# Supplementary Table 2. Risk of Bias Assessment for Included Studies (Abstracts Excluded)

| Supplementary Table 2. Risk of Bias Assessment for Included Studies (Abstracts excluded) | | | | | | | | | | | | | | | | | | | | | | | | | | | | | | | | | | | | | | | | | | | |
| --- | --- | --- | --- | --- | --- | --- | --- | --- | --- | --- | --- | --- | --- | --- | --- | --- | --- | --- | --- | --- | --- | --- | --- | --- | --- | --- | --- | --- | --- | --- | --- | --- | --- | --- | --- | --- | --- | --- | --- | --- | --- | --- | --- |
| Study | | | | |  | **Risk of Bias in Non-Randomized Studies – of Interventions (ROBINS-I) (1)** | | | | | | | | | | | | | | | | | | | | | | | | | | | | | | | | | | | | | |
|  |  |  |  |  |  | **Pre-Intervention** | | | | | | | | |  | **At Intervention** | | | | |  | | **Post-Intervention** | | | | | | | | | | | | | | | | | |  | | **Overall**  **Risk of Bias** |
|  | **Author** |  | **Year** | |  | **Bias due to Confounding** | | |  | **Bias in Selection of Participants into the Study** | | | | |  | **Bias in Selection of Classification of Interventions** | | | | |  |  | **Bias due to Deviations from intended Interventions** | | |  | **Bias due to Missing Data** | | | |  | **Bias in Measurement of Outcomes** | | | | |  | **Bias in Selection of the Reported Results** | | |  |  | **Low/**  **Moderate/**  **Serious/**  **Critical** |
| Liu et al. (2) | | | 2003 | |  | L | | |  | L | | | | |  | L | | | | |  |  | M^1^ | | |  | M^2^ | | | |  | M^3^ | | | | |  | M^4^ | | |  |  | Moderate |
| Saldaña-Cortés et al. (3) | | | 2009 | |  | L | | |  | L | | | | |  | L | | | | |  | | L | | |  | L | | | |  | M^3^ | | | | |  | L | | |  | | Moderate |
| Torres-Melero et al. (4) | | | 2016 | |  | L | | |  | L | | | | |  | L | | | | |  | | L | | |  | L | | | |  | M**^5^** | | | | |  | L | | |  | | Moderate |
|  | | | | |  | **Risk of Bias 2 (RoB 2) for Randomized Controlled Trials (5)** | | | | | | | | | | | | | | | | | | | | | | | | | | | | | | | | | | | | | |
|  |  |  |  |  |  | **Pre-Intervention** | | | | |  | | **Post-Intervention** | | | | | | | | | | | | | | | | | | | | | | | | | | | |  | | **Overall**  **Risk of Bias** |
|  |  |  |  |  |  | **Risk of Bias due to Randomization Process** | | | | |  |  | **Risk of Bias due to Deviations from intended Interventions** | | | | | |  | | **Risk of Bias due to Missing Outcome Data** | | | | | |  | **Risk of Bias in Measurement of Outcomes** | | | | | |  | | **Risk of Bias in Selection of the Reported Results** | | | | |  |  | **Low/**  **Some Concerns/**  **High** |
| Fernandez et al. (6) | | | 1996 | |  | SC^6^ | | | | |  |  | SC^8^ | | | | | |  | | SC^9^ | | | | | |  | SC^10^ | | | | | |  | | L | | | | |  |  | Some Concerns |
| Oliver et al. (7) | | | 2012 | |  | SC^7^ | | | | |  | | SC^11^ | | | | | |  | | SC^9^ | | | | | |  | SC^12^ | | | | | |  | | L | | | | |  | | Some Concerns |
| Sdralis et al. (8) | | | 2019 | |  | SC^6^ | | | | |  | | SC^13^ | | | | | |  | | SC^9^ | | | | | |  | SC^12^ | | | | | |  | | L | | | | |  | | Some Concerns |
| Silecchia et al. (9) | | | 2006 | |  | SC^6^ | | | | |  | | SC^14^ | | | | | |  | | SC^9^ | | | | | |  | SC^12^ | | | | | |  | | L | | | | |  | | Some Concerns |
| Upadhyaya et al. (10) | | | 2007 | |  | SC^7^ | | | | |  | | SC^15^ | | | | | |  | | SC^9^ | | | | | |  | SC^12^ | | | | | |  | | L | | | | |  | | Some Concerns |
|  | | | | |  | **Newcastle Ottawa Quality Assessment to assess quality of included cohort studies (out of a total of nine stars) (11)** | | | | | | | | | | | | | | | | | | | | | | | | | | | | | | | | | | | | | |
|  |  |  |  |  |  | **Selection^*^** | | | | | | | | | | | | | |  | | **Comparability^†^** | | | | | | |  | **Outcome^§^** | | | | | | | | | | |  | **Overall Quality** | |
|  |  |  |  |  |  | **A** |  | **B** | | | |  | | **C** | | |  | **D** | |  |  | **E** | |  | **F** | | | |  | **G** | | |  | | **H** | | | |  | **I** |  | **Low/**  **Moderate/**  **High**  **NOS-Score**  **(Stars, n)** | |
| Brehant et al. (12) | | | | 2013 |  | ★ |  | ★ | | | |  | | ★ | | |  | -^16^ | |  | | ★ | |  | ★ | | | |  | ★ | | |  | | ★ | | | |  | ★ |  | High (8) | |
| Huang et al. (13) | | | | 2021 |  | -^17^ |  | ★ | | | |  | | ★ | | |  | -^16^ | |  | | ★ | |  | ★ | | | |  | ★ | | |  | | ★ | | | |  | ★ |  | Moderate (7) | |
| Huh et al. (14) | | | | 2012 |  | -^18^ |  | ★ | | | |  | | ★ | | |  | -^16^ | |  | | ★ | |  | ★ | | | |  | ★ | | |  | | ★ | | | |  | -^16^ |  | Moderate (6) | |
| Kim et al. (15) | | | | 2013 |  | ★ |  | ★ | | | |  | | ★ | | |  | -^16^ | |  | | ★ | |  | ★ | | | |  | ★ | | |  | | ★ | | | |  | -^16^ |  | Moderate (7) | |
| Marano et al. (16) | | | | 2016 |  | ★ |  | ★ | | | |  | | ★ | | |  | -^16^ | |  | | ★ | |  | ★ | | | |  | ★ | | |  | | ★ | | | |  | -^16^ |  | Moderate (7) | |
| Sieda et al. (17) | | | | 2015 |  | ★ |  | ★ | | | |  | | ★ | | |  | -^16^ | |  | | -^19^ | |  | -^19^ | | | |  | -^16^ | | |  | | ★ | | | |  | -^16^ |  | Low (4) | |
| L = Low Risk; M = Moderate Risk; SC = Some Concerns  ^*^ A = Representativeness of the Exposed Cohort; B = Selection of the Non-Exposed Cohort; C = Ascertainment of Exposure; D= Demonstration that Outcome of Interest was not Present at Start of Study  ^†^ E = Comparability of cohort on the Basis o the Design and Analysis: Controlled for Critical Factor; F= Comparability of cohort on the Basis o the Design and Analysis: Controlled for Additional Factor  ^§^ G = Assessment of Outcome; H = Was the Follow Up Long Enough for Outcomes to Occur; I = Adequacy of Follow Up of Cohorts  ^1^ No air insufflation test of the anastomoses in the control group;  ^2^ Outcome data were not available for all or nearly all participants;  ^3^ No statement whether outcome assessors were aware of the intervention received by study participants;  ^4^ Bias in selection of reported result due to different subgroups  ^5^ No statement whether outcome assessors were aware of the intervention received by study participants and whether methods for outcome assessment were comparable across intervention groups;  ^6^ No description of the methods of allocation concealment;  ^7^ No statement whether allocation sequence was concealed until participants were enrolled and assigned to interventions;  ^8^ No appropriate analysis used to estimate the effect of assignment to intervention; no statement whether participants were aware of their assigned interventions during the trial; no statement whether carers and people   delivering interventions were aware of participants’ assigned intervention during the trial;  ^9^ No evidence that the results were not biased by missing outcome data and missingness in the outcome could depend on its true value;  ^10^ No statement whether measurement or ascertainment of the outcome have differed between intervention groups or whether outcome assessors were aware of the intervention received; assessment of the outcome   could have been influenced by the knowledge of intervention received;  ^11^ Carers and people delivering interventions were aware of participants’ assigned intervention during the trial; no statement whether an appropriate analysis was used to estimate the effect of assignment to intervention;  ^12^ No statement whether outcome assessors were aware of the intervention received; assessment of the outcome could have been influenced by the knowledge of intervention received;  ^13^ No statement whether participants were aware of their assigned intervention during the trial; carers and people delivering interventions were aware of participants’ assigned intervention during the trial;  ^14^ No statement whether participants were aware of their assigned intervention during the trial or whether carers and people delivering interventions were aware of participants’ assigned intervention during the   trial;  ^15^ Carers and people delivering interventions were aware of participants’ assigned intervention during the trial;  ^16^ No statement;  ^17^ Just patients undergoing McKeown esophagectomy were included, other operative procedure for the same condition were not included;  ^18^ Patients with the same procedure but with a protective stoma were excluded;  ^19^ Did not controlled for any factor using multivariate analysis or regression methods | | | | | | | | | | | | | | | | | | | | | | | | | | | | | | | | | | | | | | | | | | | |

# References

1. Sterne JA, Hernán MA, Reeves BC, Savović J, Berkman ND, Viswanathan M, et al. Robins-I: A Tool for Assessing Risk of Bias in Non-Randomised Studies of Interventions. *BMJ* (2016) 355:i4919. doi: 10.1136/bmj.i4919.

2. Liu CD, Glantz GJ, Livingston EH. Fibrin Glue as a Sealant for High-Risk Anastomosis in Surgery for Morbid Obesity. *Obes Surg* (2003) 13(1):45-8. Epub 2003/03/13. doi: 10.1381/096089203321136575.

3. Saldaña-Cortés JA, Larios-Arceo F, Prieto-Díaz-Chávez E, De Buen EP, González-Mercado S, Alvarez-Villaseñor AS, et al. Role of Fibrin Glue in the Prevention of Cervical Leakage and Strictures after Esophageal Reconstruction of Caustic Injury. *World J Surg* (2009) 33(5):986-93. Epub 2009/02/24. doi: 10.1007/s00268-009-9949-x.

4. Torres-Melero J, Motos-Micó JJ, Lorenzo-Liñán M, Morales-González Á, Rosado-Cobián R. [Use of Absorbable Fibrin Sealant Patch to Strengthen the Gastrointestinal Anastomosis Performed on Patients with Peritoneal Carcinomatosis Treated with Intention to Cure by Debulking Surgery and Intraoperative Hyperthermic Intraperitoneal Chemotherapy]. *Cir Cir* (2016) 84(2):102-8. Epub 2016/01/19. doi: 10.1016/j.circir.2015.09.005.

5. Sterne JAC, Savović J, Page MJ, Elbers RG, Blencowe NS, Boutron I, et al. Rob 2: A Revised Tool for Assessing Risk of Bias in Randomised Trials. *Bmj* (2019) 366. Epub 2019/08/30. doi: 10.1136/bmj.l4898.

6. Fernandez Fernandez L, Tejero E, Tieso A. Randomized Trial of Fibrin Glue to Seal Mechanical Oesophagojejunal Anastomosis. *British Journal of Surgery* (1996) 83(1):40-1. doi: 10.1002/bjs.1800830111.

7. Oliver JL, Medina IA, Garcia-Almenta EM, Gil JM, Sanchez MS, Diaz MDP, et al. Use of Fibrin Based Biological Adhesives in the Prevention of Anastomotic Leaks in the High Risk Digestive Tract: Preliminary Results of the Multicentre, Prospective, Randomised, Controlled, and Simple Blind Phase Iv Clinical Trial: Protissucol001. *Cirugia Espanola* (2012) 90(10):647-55. doi: 10.1016/j.ciresp.2012.05.007.

8. Sdralis E, Tzaferai A, Davakis S, Syllaios A, Kordzadeh A, Lorenzi B, et al. Reinforcement of Intrathoracic Oesophago-Gastric Anastomosis with Fibrin Sealant (Tisseel®) in Oesophagectomy for Cancer: A Prospective Comparative Study. *Am J Surg* (2020) 219(1):123-8. Epub 2019/06/27. doi: 10.1016/j.amjsurg.2019.06.013.

9. Silecchia G, Boru CE, Mouiel J, Rossi M, Anselmino M, Tacchino RM, et al. Clinical Evaluation of Fibrin Glue in the Prevention of Anastomotic Leak and Internal Hernia after Laparoscopic Gastric Bypass: Preliminary Results of a Prospective, Randomized Multicenter Trial. *Obesity Surgery* (2006) 16(2):125-31. doi: 10.1381/096089206775565249.

10. Upadhyaya VD, Gopal SC, Gangopadhyaya AN, Gupta DK, Sharma S, Upadyaya A, et al. Role of Fibrin Glue as a Sealant to Esophageal Anastomosis in Cases of Congenital Esophageal Atresia with Tracheoesophageal Fistula. *World Journal of Surgery* (2007) 31(12):2412-5. doi: 10.1007/s00268-007-9244-7.

11. Wells GA SB OCD, Peterson J et al. . The Newcastle-Ottawa Scale (Nos) for Assessing the Quality of Non Randomised Studies in Meta-Analyses (2007). Available from: [www.ohri.ca/programs/clinical_epidemiology/oxford.asp](https://d.docs.live.net/8d841b405e34154f/Desktop/TUM%20K2RANICH/External%20Coating%20anastomosis/SYNC%20%5e0%20SHARE/Supplementary%20Material/www.ohri.ca/programs/clinical_epidemiology/oxford.asp) [Accessed January 26, 2022].

12. Brehant O, Sabbagh C, Lehert P, Dhahri A, Rebibo L, Regimbeau JM. The Gentamicin-Collagen Sponge for Surgical Site Infection Prophylaxis in Colorectal Surgery: A Prospective Case-Matched Study of 606 Cases. *Int J Colorectal Dis* (2013) 28(1):119-25. Epub 2012/08/25. doi: 10.1007/s00384-012-1557-9.

13. Huang Y, Hu Y, Lin Y, Fu J, Wu J, Fang C, et al. Evaluation of Fibrin Sealant in Prevention of Cervical Anastomotic Leakage after Mckeown Esophagectomy: A Single-Center, Retrospective Study. *Annals of Surgical Oncology* (2021) 28(11):6390-7. doi: 10.1245/s10434-021-09877-0.

14. Huh JW, Kim HR, Kim YJ. Anastomotic Leakage after Laparoscopic Resection of Rectal Cancer: The Impact of Fibrin Glue. *Am J Surg* (2010) 199(4):435-41. Epub 2009/06/02. doi: 10.1016/j.amjsurg.2009.01.018.

15. Kim HJ, Huh JW, Kim HR, Kim YJ. Oncologic Impact of Anastomotic Leakage in Rectal Cancer Surgery According to the Use of Fibrin Glue: Case-Control Study Using Propensity Score Matching Method. *Am J Surg* (2014) 207(6):840-6. Epub 2013/12/10. doi: 10.1016/j.amjsurg.2013.07.047.

16. Marano L, Di Martino N. Efficacy of Human Fibrinogen-Thrombin Patch (Tachosil) Clinical Application in Upper Gastrointestinal Cancer Surgery. *J Invest Surg* (2016) 29(6):352-8. Epub 2016/05/19. doi: 10.1080/08941939.2016.1181229.

17. Sieda B, Gharib O. Comparative Study of Single-Layer Anastomosis in High-Risk Colonic Anastomosis Versus Single Layer Reinforced Using Fibrin Glue. *The Egyptian Journal of Surgery* (2015) 34:215. doi: 10.4103/1110-1121.167380.
